# A Checklist Confirming Whether a Manuscript for Submission Adheres to the Fundamentals of Academic Writing: A Proposal

**DOI:** 10.31662/jmaj.2023-0201

**Published:** 2024-02-09

**Authors:** Shigeki Matsubara, Daisuke Matsubara

**Affiliations:** 1 Department of Obstetrics and Gynecology, Jichi Medical University, Tochigi, Japan; 2 Department of Obstetrics and Gynecology, Koga Red Cross Hospital, Koga, Japan; 3 Medical Examination Center, Ibaraki Western Medical Center, Chikusei, Japan; 4 Department of Pediatrics, Jichi Medical University, Tochigi, Japan

**Keywords:** author guideline, writing style, review, reviewer, study significance

## Abstract

A manuscript written not adhering to the fundamentals of academic writing (so-called paper-writing rules) may be rejected before the significance of the study is recognized. Submitting authors, especially those with little experience, may neglect such fundamentals. A simple checklist, which would enable the authors to check whether a manuscript for submission adheres to such fundamentals, should appear at the beginning of the Author Guidelines of medical journal. This checklist may contribute to writing a manuscript following the fundamentals of academic writing, thereby preventing rejection based solely on the writing style.

## Our Proposal and Its Background

We propose that the Author Guidelines of medical journals should have a checklist through which submitting authors can confirm that they wrote the manuscript according to the fundamentals of academic writing, also referred to as fundamental paper-writing rules. Many submitted manuscripts do not adhere to such fundamentals, which causes many problems, as described later.

The fundamentals of academic writing have been established through decades-long accumulation of medical publications. Many books explain these fundamentals; they are almost the same. We based this opinion paper on the following assumptions: (i) the fundamentals of academic writing are universally accepted; (ii) manuscripts adhering to them are more readable than those that do not; and thus, (iii) medical papers, at least original articles, should adhere to them. We do not consider English language problems (spelling, words, or grammar). Here, we use the words “fundamentals of academic writing” or “fundamentals” instead of “fundamental paper-writing rules” or “rules.” Hereafter, manuscripts written adhering to these fundamentals will be referred to as “well-written manuscripts” for simplicity. There are some exceptions, which we discuss later.

## Reviewers’ Two Important Checkpoints

The first author (SM) has reviewed 2,280 manuscripts over the last 8 years (2016-2023) ^(1)^. Reviewers’ checkpoints are classified fundamentally into whether (i) the study provides some significance and (ii) the manuscript adheres to the fundamentals of academic writing. Hereafter, we refer to (i) (significance) and (ii) (adherence) as the first and second points, respectively. For judging accept or reject, the first point outweighs the second point. Experienced reviewers usually make major or general comments first and then minor or specific comments, which fundamentally correspond to the first and second points, respectively.

## A Significant Study Described in a Well-written Manuscript, and *Vice versa*

It is our experience that a significant study is almost always apparent in a well-written manuscript. A manuscript that satisfies the first point (significance) usually satisfies the second point (adherence). *Vice versa*: A manuscript that does not satisfy the first point usually does not satisfy the second point. Thus, the first and second points usually agree with each other: “yes/yes” or “no/no.” The second point requires less time for reviewers to judge than the first point; the second point is easier to discern than the first point. For reviewers, “not well written” is often a sign of a study with little significance ^(2)^. Because experienced reviewers immediately discern the second point and because a manuscript with a low second point score usually does not involve significance worthy of publication (first point), a not well-written manuscript is often rejected without being carefully evaluated regarding the first point; the review proceeds from the second to the first point, which is contrary to the fundamental review policy: the first (significance) and the second point (how to write) comes first and second, respectively.

## Possibility of Overlooking Manuscripts Describing a Significant Study

We reviewers likely overlook a significant study described in a poorly written manuscript. Not all show either yes/yes or no/no. Although rare, there are some manuscripts with “yes/no”; the study involves significance worthy of publication, but the manuscript is not well written. Recognizing such manuscripts is difficult even for experienced reviewers. How pleasant it would be if all submitted manuscripts fundamentally adhered to the fundamentals of academic writing! Thus, we have a proposal.

## Our Proposal: An Example

We propose that a journal should provide a checklist focusing on “how to write.” Table 1 ^(3)^ presents an example of a checklist. This checklist should appear at the beginning of the Author Guidelines. Writing the Methods and Results depends on the study design. Thus, we omit it.

**Table 1.** Our Proposal of an “Author Checklist” that Enables Authors to Confirm Whether a Manuscript for Submission Adheres to the Fundamentals of Academic Writing.

Please check the following items:	
(1)	The title is informative ^(3)^. The title involves the major findings or at least study targets or methods, or both. The title does not include the words “study on,” “research into,” or “analysis of,”
(2)	The Introduction consists of three sections: (i) known, (ii) unknown, and (iii) problem and (i) what is already known, (ii) what remains unknown, and (iii) what has been attempted to clarify here. If the subject is narrow, (ii) and (iii) can be combined.
(3)	The end of the Introduction consists of a summary of (iii); what is the problem or question to be clarified or answered.
(4)	The first paragraph of the Discussion consists of a short direct answer to the Introduction (iii), i.e., “what has been clarified or answered.”
(5)	Study limitations appear in the second paragraph from the last paragraph of the Discussion.
(6)	The last paragraph of the Discussion involves the study’s significance.
Authors are recommended to adhere to this list as much as possible. Editors of the journal understand that some authors have their own writing style. Nonadherence to this checklist does not become a reason for rejection.	

This checklist should reflect the journal policy. For example, some journals permit an “indicative title” or a “suggestive title” (a title indicating the study theme) ^(3)^. Then, please delete item (1) in Table 1. Some journals have a policy to place study limitations in the second (not second from the last) paragraph of the Discussion. Then, please indicate so in item (5) in Table 1. Thus, the Table 1 is only an example to facilitate an open discussion.

## A Checklist Beneficial for All

This checklist is beneficial for all: including authors, reviewers, and journals. First, it is beneficial for authors. The study has already been completed. Thus, the authors cannot fundamentally change the study’s significance. However, how to write the manuscript can be modified; the manuscript can become better written. If one does not understand an item, such as item (2), one should read a textbook chapter on how to write a good Introduction ^(4), (5)^. This focused reading may require less time than reading an entire textbook.

Second, the checklist benefits reviewers. If well written, reviewers can focus more on evaluating the first point: the study’s significance. We need not repeatedly write in a review sheet: “Please delete “study on” from the title” or “Please write your clinical question (problem) at around the end of the Introduction.”

Third, journals and medical and scientific societies may also benefit from the checklist. A significant study may be less likely to become buried based solely on the judgment of being poorly written. Reviewers and journal editors may be able to help make the manuscript better if it is just a matter of the writing style.

## Problems and Future Directions

We wish to discuss some problems and future directions relating to the present issue. First, thanks to this checklist, what if all submitted manuscripts pass the second point? To judge a no/yes type manuscript (little significance but well written), like judging a yes/no one (significant but not well written), we reviewers must have keener eyes ^(2)^. Reviewers’ responsibilities may increase.

Second, some experienced writers produce a readable and beautiful manuscript, although it does not adhere to the fundamentals of academic writing. They have their own style. This type of manuscript should be acceptable. However, whether it is perceived beautiful depends on the individual reviewer’s feelings.

Third, medical societies, journals, and older doctors should nurture good writers. The submitting authors will use this checklist at the time of submission. Although we believe that this checklist will help nurture good writers, we must consider some effective systems for nurturing good next-generation writers.

Fourth, artificial intelligence involving chatbots, such as ChatGPT, may strongly affect this issue ^(2)^. We must question whether and how chatbots affect medical paper writing and how to deal with it, which must be considered carefully.

## What Is a Well-written Manuscript?

When one has a conviction and is eager to express one’s belief, one may be able to write it persuasively. Someone talented in writing may write highly readable papers. JAMA publishes medical poems, which always impress us. Thus, adherence to the fundamentals of academic writing is not mandatory. However, most authors are not very talented. Thus, it is safer and more innocuous for them to adhere to the fundamentals of academic writing. We hope our present proposal may help improve medical writing and may arouse broad discussions regarding this proposed list and medical paper writing in general.

## Article Information

### Conflicts of Interest

None

### Author Contributions

SM wrote the manuscript. DM edited the manuscript.

### Approval by Institutional Review Board (IRB)

Not applicable.

### Data Availability

Data sharing does not apply to this article because no new data were created or analyzed in this study.

### Patient Anonymity

Not applicable.

### Informed Consent

Not applicable.
